# Construction and expression of a human/mouse chimeric CD19 monoclonal antibody: Successful modification of a murine IgM to a chimeric IgG

**DOI:** 10.3892/etm.2014.1511

**Published:** 2014-01-29

**Authors:** WEIQUN XU, LINGYAN ZHANG, YI ZHANG, YONGMIN TANG

**Affiliations:** 1 Department of Hematology and Oncology, School of Medicine, Children’s Hospital of Zhejiang University, Hangzhou, Zhejiang 310003, P.R. China; 2 Key Laboratory, The First Hospital of Ningbo City, Ningbo, Zhejiang 315700, P.R. China

**Keywords:** CD19, chimeric antibody, baculovirus shuttle vector, antibody engineering, leukemia

## Abstract

CD19 is a specific surface marker of B cells. A murine IgM-subtype antibody, 2E8, was generated previously and assigned to the CD19 category by the 6th International Workshop and Conference on Human Leukocyte Differentiation Antigens in 1996. In the present study, the 2E8 Fv gene was inserted into a baculovirus shuttle vector and novel protein was expressed in an IgG1 form in the Sf9 insect cell line. VH_2E8_ and VL_2E8_ genes were cloned and inserted into the baculovirus shuttle vector pAc-κ-CH3 to form pAc-κ-CH3-VH_2E8_-VL_2E8_. Sf9 cells were then transfected with the reconstructed baculovirus shuttle vector. Novel protein expressed by the Sf9 cells was identified by immunofluorescence and western blot analysis, while activity levels were analyzed by flow cytometry (FCM). Sequencing demonstrated that the VH_2E8_ and VL_2E8_ fragments were inserted into pAc-κ-CH3 correctly. The immunofluorescence, western blot analysis and FCM results indicated that active recombinant antibody was expressed in the cytoplasm of Sf9 cells, but not in the culture supernatant. Thus, functional recombinant antibody was expressed successfully in the cytoplasm of Sf9 cells, but was not secreted into the culture supernatant. Therefore, the present study demonstrates that it is possible to modify mouse IgM to mouse-human chimeric IgG1 while retaining reasonable biological activity.

## Introduction

B-lineage acute lymphoblastic leukemia is the most common type of acute leukemia and remains a major life-threatening disease in children (1). Compared with conventional chemotherapy, targeted monoclonal antibody (mAb) therapy is more effective and has fewer side-effects due to greater selectivity and specificity (2–6). mAbs directed against CD molecules on the surface of leukemic cells have shown promising results for targeted therapy (7). CD19 is a specific surface marker of B cells and is consistently and steadily expressed in almost all the differentiation stages of B-lineage cells (8). Therefore, CD19 may be an ideal target for the development of antibody-based treatments for B-lineage malignancies (9–11).

Currently, the majority of high-affinity mAbs available are of mouse origin. The clinical application of mouse antibodies has been greatly restricted due to the occurrence of the human anti-mouse antibody response, which decreases the efficacy of therapy and may cause severe anaphylaxis (12,13). Thus, it is necessary to reduce the immunogenicity of mouse antibodies, but maintain maximal antigen-recognizing activity. A successful model of a chimeric antibody used in targeted therapy in combination with chemotherapy is rituximab (human-mouse chimeric anti-human-CD20 antibody). Treatment with rituximab has improved the efficacy of B-lineage non-Hodgkin lymphoma therapy by 40% (3). However, the majority of B-lineage leukemic cells are immature and lack expression of CD20 on the cell surface, restricting the application of rituximab in leukemia treatment. By contrast, CD19 is expressed at various differentiation stages of B lymphocytes, from stem cells to mature B cells, throughout B-lineage leukemia. Therefore, CD19 is an improved target molecule for B-lineage leukemia therapy (8).

The baculovirus expression system is widely applied as an important eukaryotic expression system due to a number of advantages, including the ease of inserting desired genes, high yield, the presence of post-translational modifications similar to those found in human cells, ease of use and suitable biological material safety. The baculovirus expression vector pAc-κ-CH3 was designed particularly for the expression of chimeric antibodies; it comprises IgG expression cassette elements, including authentic IgGκ and heavy chain signal sequences, as well as light chain κ and the heavy chain constant region, that are integrated into a single vector and controlled by p10 and polyhedrin promoters, respectively (14). IgG yields have been reported to be between 6 and 18 mg/l and the antigen-binding function has been observed to be well-preserved (15). Therefore, this system was selected for the expression of recombinant protein in the present study. However, modifying IgM to IgG is likely to be a difficult procedure since changing the pentameric IgM unit to a dimeric IgG unit may result in the loss of binding activity (16,17).

Zhejiang Children’s Hospital (ZCH)-4-2E8 or simply 2E8, is an antibody belonging to the murine IgM subtype that was generated in the Department of Hematology and Oncology, School of Medicine, Children’s Hospital of Zhejiang University (Hangzhou, China) (18). 2E8 was assigned to the CD19 category by the 6th International Workshop and Conference on Human Leukocyte Differentiation Antigens (HLDA6) in 1996. The aim of the present study was to insert 2E8 Fv genes into the pAc-κ-CH3 baculovirus shuttle vector and express the novel protein in the insect Sf9 cell line, using the baculoviral expression system. The characteristics and physiological activity of the chimeric antibody were also examined.

## Materials and methods

### Cell culture

Experimental procedures were approved by the Medical Ethics Committee of the Children’s Hospital of Zhejiang University.

The Sf9 insect cell line was provided by Professor Mifang Liang from the Chinese Center for Disease Control Prevention, Institute for Viral Disease Control and Prevention (Beijing, China) (15). Sf9 cells were cultured in serum-free medium (SFM; SF900 II; Invitrogen Life Technologies, Shanghai, China) and fetal bovine serum (Gibco-BRL, Gaithersburg, MD, USA), but were gradually adapted to SFM prior to the expression studies. NALM-6, a B-lineage leukemic cell line, was cultured in RPMI-1640 medium (Gibco-BRL) and fetal bovine serum.

The hybrid cell line 2E8, which secreted the parental 2E8 anti-human CD19 mAb, was cultured in RPMI-1640 medium and fetal bovine serum.

### Plasmids and reagents

**Figure 1:**
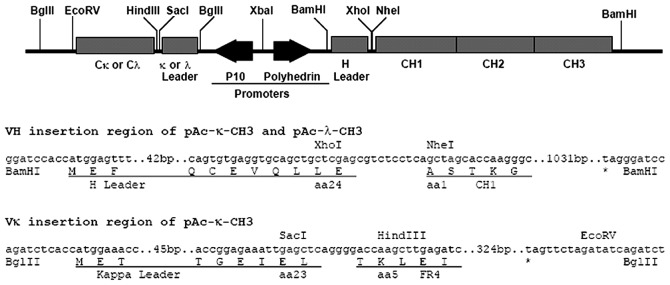
Structure of the pAc-κ-CH3 baculovirus expression vector.

The Psectag2A/Scfv2E8 plasmid, which contained heavy and light chains of the 2E8 mAb, was established in the Department of Hematology and Oncology, School of Medicine, Children’s Hospital of Zhejiang University. Psectag2A, *Escherichia coli* (*E. coli*) DH5α strain cells, high-fidelity platinum *Taq* polymerase, *Taq* DNA polymerase, RQ1 5-bromo-4-chloro-indolyl-β-D-galactopyranoside, isopropylthio-β-galactoside, RNasin and RNase-free DNase were purchased from Invitrogen Life Technologies. The pAc-κ-CH3 baculovirus expression vector, which contained authentic IgGκ, heavy chain signal sequences and constant regions, was provided by Professor Mifang Liang from the Chinese Center for Disease Control Prevention, Institute for Viral Disease Control and Prevention (15). The structure of this plasmid is shown in Fig. 1 (15).

pGEM^®^-T Easy Vector (TA cloning) and the restriction endonucleases, *Eco*RI, *Sac*I, *Hin*dIII, *Xho*I, *Nhe*I, *Bam*HI and *Bgl*I, were purchased from Promega Corporation (Beijing, China). The BaculoGold transfection kit, mouse anti-human (MAH) γ1-fluorescein isothiocyanate (FITC) and dialysis solution were purchased from Becton Dickinson (Franklin Lakes, NJ, USA). Horseradish peroxidase (HRP)-conjugated MAH-Fc-HRP antibodies were purchased from Sigma-Aldrich (Shanghai, China). Goat anti-mouse (GAM)-Fab-rhodamine, HRP-conjugated GAM-μ-HRP and GAM-Fab-HRP antibodies were purchased from Rockland Immunochemicals, Inc. (Gilbertsville, PA, USA). MAH-Fc-FITC, GAM-Fab-(κ)-FITC and SuperSignal West Durab Extended Duration Substrate were purchased from Pierce Biotechnology, Inc. (Rockford, IL, USA). T4 DNA ligase and Triton X-100 were purchased from Gibco-BRL and a DL2000 marker was purchased from Takara Bio, Inc. (Dalian, China). A QIAquick gel extraction kit was purchased from Qiagen (Valencia, CA, USA) and the SuperFect transfection reagent was purchased from Roche Diagnostics (Shanghai, China). Oligo(dT)_12–18_ primers and polyvinylidene fluoride (PVDF) membranes were purchased from Bio-Rad Laboratories, Inc. (Shanghai, China). A prestained protein molecular weight marker was purchased from Fermentas, (Shenzhen, China) and HRP-labeled GAM IgG (heavy and light chains) was purchased from Beijing Zhongshan Golden Bridge Biotechnology Co., Ltd. (Beijing, China).

### Construction of the pAc-κ-CH3-VH_2E8_-VL_2E8_ baculovirus shuttle vector

VH_2E8_ and VL_2E8_ genes were cloned from pSectag2A/ScFv_2E8_, which had been successfully established previously by polymerase chain reaction amplification using the primers listed in Table I. Specific endonuclease sites were located within the primer pairs and the amplified fragments were inserted using TA cloning techniques. This was followed by transformation into *E. coli* DH5α cells. Recombinants were selected and amplification and sequencing of the inserted sequences were performed. Target sequences were confirmed by comparison with the previously cloned VH_2E8_ and VL_2E8_ gene sequences to enable further study.

VH_2E8_ and VL_2E8_ gene fragments were cleaved with corresponding endonucleases (VH_2E8_, *Xho*I and *Nhe*I; VL_2E8_, *Sac*I and *Hin*dIII) and inserted sequentially into the secretive pAc-κ-CH3 baculovirus expression shuttle vector. Following transformation into *E. coli* DH5α cells, recombinants were selected, plasmid DNA was purified and the insertions were amplified and sequenced using the method described by Liang *et al* (14). The sequences were then compared with the previously identified VH_2E8_ and VL_2E8_ gene sequences to confirm that the insertions were correct. DNA manipulation and bacterial transformation procedures were conducted as previously described by Filpula *et al* (16).

### Transfection of Sf9 cells with the reconstructed baculovirus shuttle vector and the formation of the pAc-κ-CH3-VH_2E8_-VL_2E8_ complete virion (CV)

Recombinant baculoviruses were prepared by homologous recombination using the BaculoGold transfection kit (Becton Dickinson, Franklin Lakes, NJ, USA), according to the manufacturer’s instructions. Sf9 cells were cotransfected with the pAc-κ-CH3-VH_2E8_-VL_2E8_ reconstructed shuttle vector and linearized DNA of the *Autographa california* nuclear polyhedrosis virus (AcNPV). pXyIE and AcNPV linearized DNA-transfected Sf9 cells and uninfected Sf9 cells were set as positive and negative controls, respectively, as recommended by the manufacturer’s instructions. Morphological changes in the cells were observed every day following transfection using an inverted microscope. Positive control cells expressing recombinant XyIE turned yellow in the presence of catechol at day 4 following transfection. The supernatants of the pAc-κ-CH3-VH_2E8_-VL_2E8_-transfected Sf9 cells were harvested as primary recombinant CVs, to produce pAc-κ-CH3-VH_2E8_-VL_2E8_ CV (P0) for further amplification. Transfected Sf9 cells were collected for detection on day 7.

Through three passages of amplification, large viral stocks were prepared by infecting Sf9 cells at a multiplicity of infection (number of virions/number of cells being infected) of <1. The supernatant was harvested at day 4 or 5 following infection. Three passages were amplified and the virus stock was saved for application in the expression studies.

For protein expression, Sf9 cells were cultured in SFM. The supernatant was collected for detection at day 6 following infection when ~30% of living cells remained.

### Identification of the recombinant protein by flow cytometry (FCM)

To analyze the activity levels of the recombinant antibody in the supernatant and cell lysates, a 1×10^6^ cells/tube suspension of fresh NALM-6 cells was prepared in six tubes. Next, 100 μl concentrated expression supernatant or infected Sf9 cell lysate was added to the cell suspension in two of the tubes and the same volume of concentrated regular medium (each in duplicates) was added to the other four tubes as negative controls. After 30 min, the cells were washed twice with phosphate-buffered saline (PBS). MAH-Fc-FITC and GAM-κ-FITC were added separately and the reactions were incubated for 30 min, which was followed by two washes with PBS. FCM analysis was utilized to observe whether the chimeric antibody in the supernatant or infected Sf9 cell lysate was able to bind to the CD19 antigen on the NALM-6 cell surface.

### Identification of the recombinant protein by western blot analysis

Sf9 cells (2×10^7^ cells) were placed in 1 ml lysis refolding solution [50 mmol/l Tris-HCl (pH 7.5), 50 mM NaCl, 5 mM oxidized glutathione, 0.5 mM reduced glutathione and 1 M urea] with 100 mM phenylmethylsulfonyl chloride, 1 μg/ml aprotinin and 1 μg/ml leupeptin to prevent protein degradation. Cells were sonicated to obtain the cell lysate and pAc-κ-CH3-VH_2E8_-VL_2E8_ CV P3-infected Sf9 cells were set as the experimental group and the uninfected Sf9 cells lysates were used as the negative control. Lysates were dialyzed in PBS (containing 0.02% sodium azide) for 48 h (used PBS was replaced with fresh PBS six times in this duration), following which PBS substitution ultrafiltration was performed twice. Protein concentration was determined using the DC protein assay kit (Bio-Rad Laboratories, Inc.). Total protein extracts (0.5 μg/well) were separated by sodium dodecyl sulfate-polyacrylamide gel electrophoresis (SDS-PAGE), according to the method described by Sambrook *et al* (16), using 12% separation polyacrylamide gels and 5% condensed gels. Gels were stained with Coomassie brilliant blue R250 (16). For western blot analysis, the chimeric antibody was transferred onto PVDF membranes and blocked with 5% skimmed milk. The transferred membrane was incubated with MAH-Fc-HRP (1:20,000) for 2 h at 37°C and washed three times. Detection was performed using an enhanced chemiluminescence substrate and exposure to X-ray film. The anti-human CD20 mouse-human chimeric antibody, rituximab (Roche/Genentech, San Francisco, CA, USA), was used as a positive control.

## Results

### Construction of the pAc-κ-CH3-VH_2E8_-VL_2E8_ recombinant baculovirus shuttle vector

The 380-bp VH_2E8_ and 330-bp VL_2E8_ gene fragments were amplified using the primers listed in Table I and inserted into the TA-cloning vector. Endonuclease digestion, sequencing and sequence analysis were then performed. The correct sequences were named TA-VH_2E8_ and TA-VL_2E8_.

TA-VH_2E8_ and pAc-κ-CH3 vectors were digested with *Xho*I and *Nhe*I and ligated with T4 DNA ligase. The recombinant was treated with *Bam*HI endonuclease and sequenced to confirm the correct insertion and orientation of recombinant pAc-κ-CH3-VH_2E8_. The VL_2E8_ fragment was inserted into pAc-κ-CH3-VH_2E8_, which was confirmed by sequencing.

### Transfection, virion amplification and expression of the recombinant antibody

**Figure 2:**
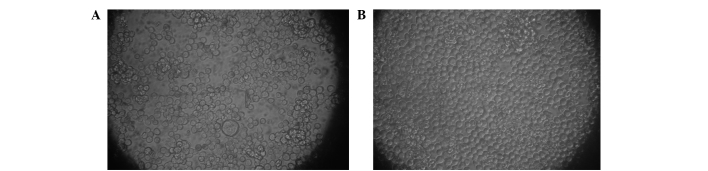
Morphology of (A) Sf9 cells infected with pAc-κ-CH3-VH_2E8_-VL_2E8_ and (B) uninfected Sf9 cells (negative control) at day 4 following infection. The infected cells have stopped proliferating and are various sizes and irregular shapes. Certain cells are larger with increased intracellular granules, and >5% of the cells are in suspension with a small amount of cell debris present. Uninfected cells are of uniform size, rounded shape and have good cell permeability. There is no cell debris and <2% of the cells are in suspension, indicating that the cells are healthy.

**Figure 3:**
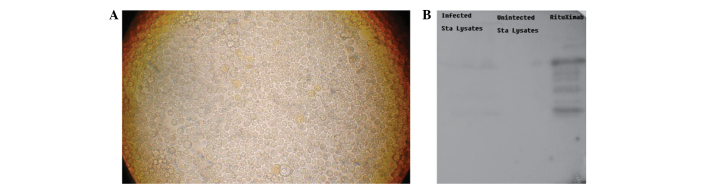
(A) Morphology of Sf9 cells transfected with the pXyIE positive control plasmid. Infected cells have various sizes and irregular shapes. Certain cells are larger with increased intracellular granules and >5% of the cells are in suspension with a small amount of cell debris. Cells were harvested and 5–10% of the cells were stained bright yellow in the presence of catechol (day 4 following infection), indicating successful transfection. (B) Western blot analysis. Lanes 1, cell lysates from infected Sf9 cells cultured in SFM with heavy and light chains (positive control); 2, uninfected Sf9 cell lysates have no bands corresponding to the heavy and light chains; and 3, rituximab has bands corresponding to the heavy and light chains. SFM, serum-free medium.

Sf9 cells were transfected with the pAc-κ-CH3-VH_2E8_-VL_2E8_ baculovirus expression vector and transfected cells were identified to be bigger and of irregular shape (Fig. 2A) when compared with the negative control cells (Fig. 2B). The positive control cells turned yellow in the presence of catechol when harvested at day 4 following transfection (Fig. 3A), indicating that the transfection procedure was successful.

Following three passages of amplification, high-titer virus stocks were ready for use in the expression studies.

### Determination of recombinant protein activity levels by FCM

Antibody activity was detected using FCM in the cell lysates from infected Sf9 cells, but not in the supernatant.

**Figure 4:**
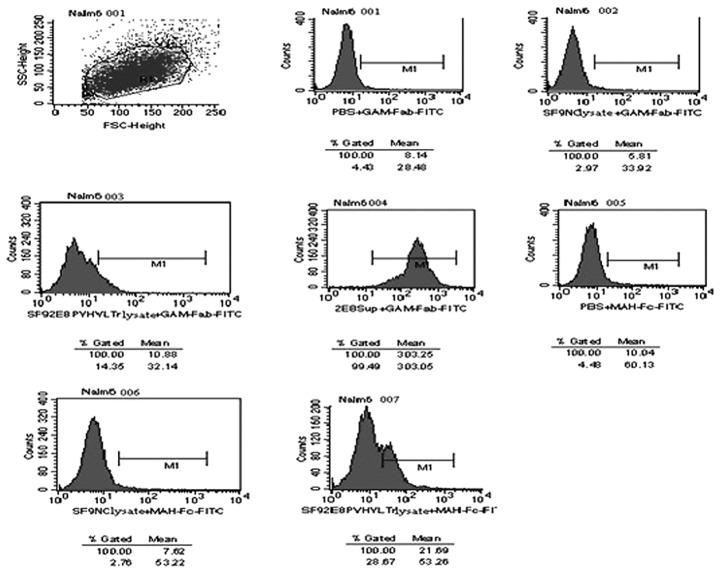
Detection of antibody activity levels in the cell lysates of infected Sf9 cells using an indirect immunofluorescence test. Cell lysates of Sf9 cells infected with pAc-κ-CH3-VH_2E8_-VL_2E8_ were added to NALM-6 cells and then incubated with FITC-labeled MAH-Fc and GAM-Fab. The percentage of positive cells in the GAM-Fab-FITC group was 14.35% compared with 2.97% in the negative control where NALM-6 cells were treated with uninfected Sf9 cell lysates. The percentage of positive cells was 28.67% in the MAH-Fc-FITC group, indicating that active recombinant antibody was present in the infected cell lysates. MAH, mouse anti-human; GAM, goat anti-mouse; FITC, fluorescein isothiocyanate.

NALM-6 cells, incubated with the cell lysates from infected Sf9 cells, were 14.35% positive (vs. 2.97% in the negative control) when labeled with GAM-Fab-FITC. The percentage of positive cells (28.67 vs. 2.76% in the negative control) was even higher when labeled with MAH-Fc-FITC, which indicated the existence of functional antibody in the infected Sf9 cell lysates (Fig. 4).

### Identification of the recombinant protein by immunofluorescence and western blot analysis

**Figure 5:**
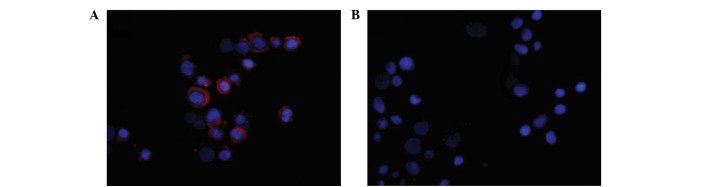
Detection of the reconstructed antibody using GAM-Fab-rhodamine immunofluorescence in (A) Sf9 cells infected with pAc-κ-CH3-VH_2E8_-VL_2E8_ and (B) uninfected Sf9 cells at day 7 following infection. In the infected group,80% of the cells were positive (red) for the GAM-Fab-rhodamine fluorescence signal. GAM, goat anti-mouse.

Since Sf9 cells exhibited green autofluorescence, immunofluorescence in the transfected Sf9 cells was monitored by incubation with GAM-Fab-rhodamine instead of FITC. It was found that 80% of the cells were positive for red fluorescence in the cytoplasm (Fig. 5A), indicating the expression of novel protein in the cytoplasm of infected Sf9 cells. However, no fluorescence was observed in the uninfected Sf9 cells (Fig. 5B).

No specific protein bands corresponding to the heavy and light chains were observed in the supernatant from the infected cells. However, specific bands were observed in the lysates of the infected Sf9 cells, which was consistent with the FCM results. Specific bands corresponding to the heavy and light chains of rituximab were observed by western blot analysis in the infected Sf9 cell lysate, while the uninfected Sf9 cell lysates did not exhibit any positive activity (Fig. 3B).

## Discussion

There are five main classes of Ig: IgG, IgA, IgD, IgE and IgM, divided according to the various genes encoding the constant regions of the heavy chain. IgG and IgA classes are in turn subdivided into six isotypes: IgG1, IgG2, IgG3, IgG4, IgA1 and IgA2. When reconstructing expression vectors for antibodies, it is generally accepted that IgGs, including IgG1, IgG2 and IgG4 subtypes, may be genetically manipulated with relative ease whilst maintaining antibody function. In total, >20 recombinant IgG antibody-based therapeutic drugs are now licensed for the treatment of a variety of diseases, the majority of which belong to the IgG1 subclass. In addition, there are hundreds of new drugs currently under development (9). In the present study, a vector with the IgG1 form of the final product was selected to express a chimeric antibody. It was previously reported that difficulties may arise when transforming IgM into IgG (17). Thus, the outcome of the CD19 antibody in the current study was not clear. Genetic manipulation was performed on ZCH-4-2E8, an IgM-type antibody developed previously and identified to be a novel CD19 mAb by HLDA6 in 1996. Gene cloning of the variable regions of the heavy and light chains of this antibody was successfully performed and a eukaryotic expression system was constructed. Following the insertion of the genes encoding the chimeric antibody into the pAc-κ-CH3 baculovirus vector and transfection into the insect host Sf9 cell line, the antibody was shown to be expressed in the cytoplasm by immunostaining with a rhodamine-labeled GAM-Fab antibody. This was further confirmed by SDS-PAGE and western blot analysis. The results obtained indicate that genes encoding the IgM variable region may be reconstructed with an IgG backbone and retain part of the antibody function. In addition, the results demonstrate that specific IgM antibodies may also be amenable to genetic manipulation and are likely to retain part of the capacity to recognize antigens.

FCM was applied to detect the activity levels of the recombinant antibody. Activity was detected in the lysates, however, the levels were reduced compared with those of the parental antibody. In addition, activity was not detected in the supernatants. These results indicate that the antibody was produced inside the cells, but was unable to be secreted outside the cells. The results indicate that it was possible to modify IgM into an IgG form and partially retain its activity. However, questions remain with regard to promoting the secretion of the engineered antibody, while retaining or improving its biological functions. Correct antibody conformation is critical to biological function, including the recognition of the relevant antigen. It remains to be investigated whether transformation from IgM to IgG causes changes in the spatial conformations of the chimeric antibody that prevent secretion or whether the leader sequence of the IgG form is not suitable for the secretion of the IgM variable region. Therefore, further studies are required to engineer antibodies that are able to be secreted from cells, to improve the binding activity of the antibodies and to identify appropriate leader sequences that promote efficient secretion, while retaining optimal biological function via modulation of the gene sequences.

In the present study, the recombinant shuttle vector, pAc-κ-CH3-VH_2E8_-VL_2E8_, was successfully reconstructed and CVs with the capacity for natural infection of insect cells were obtained. Recombinant antibody was successfully expressed and functional antibody existed inside the infected Sf9 cells but was not secreted. Therefore, the results indicate that it is possible to reconstruct an IgG-form chimeric antibody from a parental IgM antibody. However, modifications of the procedure are required to obtain a secreted chimeric antibody with appropriate biological activity.

## Figures and Tables

**Table I:** Primers used to clone VH_2E8_ and VL_2E8_ genes for insertion into pAc-κ-CH3.

Gene	Up/down	Sequence
VH_2E8_	Forward	CTCGAGGAGGTGAAGCTGGTGGAGT (*Xho*I)
VH_2E8_	Reverse	GCTAGCCTCTGAGGAGACGGTGACT (*Nhe*I)
VL_2E8_	Forward	GAGCTCGATATCCAGATGACACAGACTTC (*Sac*I)
VL_2E8_	Reverse	AAGCTTTTTGATTTCCAGCTTGGTGCCT (*Hin*dIII)
